# Supplementary material for: Identification of Freezing-Responsive microRNAs and Their Targets in Chinese Jujube by Small RNA and Degradome Sequencing
Source: Int J Mol Sci. 2026 Sep 4;27(17):7912. doi: 10.3390/ijms27177912 (PMC13566890; doi:10.3390/ijms27177912)
Supplement: Supplementary file 1 [file ijms-27-07912-s001.zip › Supplementary Material S2/novel_miR_57.pdf]

Provisional ID : novel_miR_57
Score total : 900.9
Score for star read(s) : 3.9
Score for read counts : 894.4
Score for mfe : 1
Score for randfold : 1.6
Score for cons. seed :
Total read count : 1766
Mature read count : 1748
Loop read count : 0
Star read count : 18

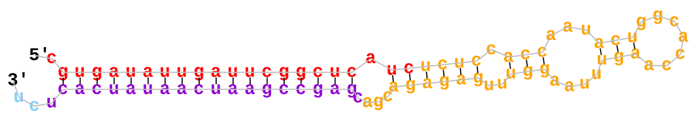

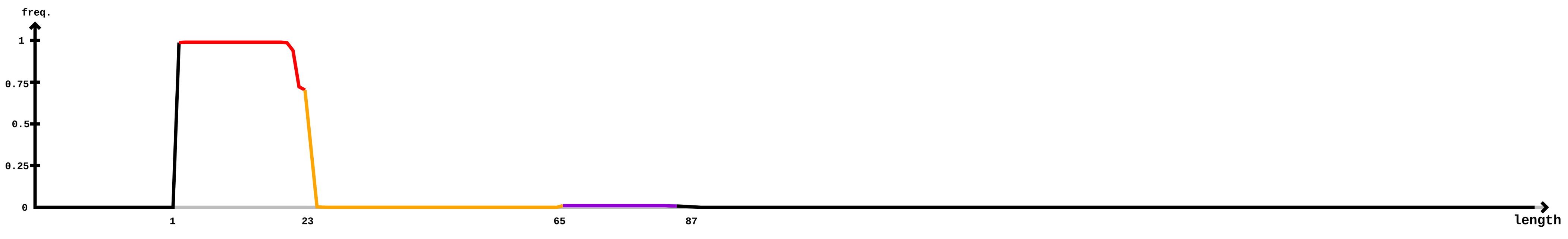

Mature Star

```
5'- cauucugcagaaguagacauggcgugauauugauucggcucaucucuccaccaauacuggcaccaaguuuaagguuugagagacgacgagccgaaucaauaucacucuuguaugcuucuucgcauauuaucaagguuuuuccuguuuuuugaguuccaauauuagccaagaagcaucucugcaggcaacucaugcauuaagcuacauagaagcuuguuuuggcucuccaucccccucuuguaaagucuuc  -3'  obs
    cauucugcagaaguagacauggcgugauauugauucggcucaucucuccaccaauacuggcaccaaguuuaagguuugagagacgacgagccgaaucaauaucacucuuguaugcuucuucgcauauuaucaagguuuuuccuguuuuuugaguuccaauauuagccaagaagcaucucugcaggcaacucaugcauuaagcuacauagaagcuuguuuuggcucuccaucccccucuuguaaagucuuc        exp
    ....(((((((........((.(((((((((((((((((.((((((.(((...(((.......)))....)))..))))))....))))))))))))))))).)).((((((......))))))......((.....))(((((((((.(((........))))))))))))..)))))))(((.....)))..((((((.......))))))....((((...((.........))...))))...   reads  mm  sample
    ......................cgugauauugauucggcu............................................................................................................................................................................................................   1      0   LT-1
    ......................cgugauauugauucggcu............................................................................................................................................................................................................   5      0   LT-3
    ......................cgugauauugauucggcuc...........................................................................................................................................................................................................   7      0   LT-1
    ......................cgugauauugauucggcuc...........................................................................................................................................................................................................   13     0   LT-3
    ......................cgugauauugauucggcuc...........................................................................................................................................................................................................   33     0   LCK-3
    ......................cgugauauugauucggcuU...........................................................................................................................................................................................................   1      1   LT-3
    ......................cgugauauugauucggcuc...........................................................................................................................................................................................................   21     0   LT-2
    ......................cgugCuauugauucggcuc...........................................................................................................................................................................................................   1      1   LCK-3
    ......................cgugauauugauucggcuA...........................................................................................................................................................................................................   6      1   LCK-3
    ......................cgugauauugauucggcuca..........................................................................................................................................................................................................   2      0   JCK-1
    ......................cgugauCuugauucggcuca..........................................................................................................................................................................................................   1      1   LCK-1
    ......................cgugauCuugauucggcuca..........................................................................................................................................................................................................   1      1   LT-3
    ......................cgugauauugauucggcucU..........................................................................................................................................................................................................   3      1   LT-2
    ......................cgugauCuugauucggcuca..........................................................................................................................................................................................................   1      1   LCK-2
    ......................cgugauauugauucggcuca..........................................................................................................................................................................................................   71     0   LT-2
    ......................cgugauauugauucggcuca..........................................................................................................................................................................................................   60     0   LCK-2
    ......................cgugauauugauucggcuca..........................................................................................................................................................................................................   52     0   LT-3
    ......................cgugauauugauucggcuca..........................................................................................................................................................................................................   1      0   JT-2
    ......................cgugauauugauucggcuca..........................................................................................................................................................................................................   48     0   LCK-1
    ......................cgugauauugauucggcuca..........................................................................................................................................................................................................   9      0   JCK-2
    ......................cAugauauugauucggcuca..........................................................................................................................................................................................................   1      1   LT-2
    ......................cgugauauugauucggcuca..........................................................................................................................................................................................................   3      0   JCK-3
    ......................cgugauauugauucggcuca..........................................................................................................................................................................................................   103    0   LCK-3
    ......................cgugauauugauucggcuca..........................................................................................................................................................................................................   3      0   JT-3
    ......................cgugauauugauucgAcuca..........................................................................................................................................................................................................   1      1   LCK-3
    ......................cgugauauugauucggcuca..........................................................................................................................................................................................................   23     0   LT-1
    ......................cgugauCuugauucggcucau.........................................................................................................................................................................................................   1      1   LT-3
    ......................cgugauauugauucggcucau.........................................................................................................................................................................................................   14     0   LCK-3
    ......................cgugauauugauucggcucaA.........................................................................................................................................................................................................   1      1   LT-3
    ......................cgugauauugauucggcucau.........................................................................................................................................................................................................   8      0   LT-1
    ......................cgugauauugauucggcucau.........................................................................................................................................................................................................   1      0   LT-3
    ......................cgugauauugauucggcucau.........................................................................................................................................................................................................   9      0   LT-2
    ......................cgugauauugauucggcucauU........................................................................................................................................................................................................   1      1   JCK-2
```

Mature / **Star**

cauucugcagaaguagacaugg**cgugauauugauucggcucauc**ucuccaccaauacuggcaccaaguuuaagguuugagagacga**cgagccgaaucaauaucacu**cuuguaugcuucuucgcauauuaucaagguuuuuccuguuuuuugaguuccaauauuagccaagaagcaucucugcaggcaacucaugcauuaagcuacauagaagcuuguuuuggcucuccauccccccucuuguaaagucuuc

cgugauauugauucggcucauU	3	1	LT-1
cgugauauugauucggcucauc	31	0	LCK-2
cgugauauugauucggcucCuc	1	1	LT-2
cgugauauugauucggcucauc	31	0	LCK-1
cgugauauugauucggcucauc	248	0	LT-3
cgugauauugCuucggcucauc	1	1	JCK-1
Agugauauugauucggcucauc	1	1	LT-1
cgugauauugCuucggcucauc	1	1	LT-1
cgugauauugauucggcucauc	51	0	JCK-1
cgugauauugauucggcucauc	237	0	LT-1
cgugauauugauucggcucauU	9	1	LCK-3
cgugauauugauucggcucauc	122	0	LT-2
cguUauauugauucggcucauc	1	1	LT-2
cguAauauugauucggcucauc	1	1	JCK-3
cgugauauugauucggcucauc	99	0	JCK-2
cgugCuauugauucggcucauc	1	1	LCK-3
cgugauauugauucggcucauU	5	1	LT-3
cgugauauugauucggcCcauc	1	1	LT-1
cgGgauauugauucggcucauc	1	1	LT-2
cgugauauugauucggcucauU	5	1	JCK-3
cgugauauugauucggcucauc	93	0	JCK-3
cgugCuauugauucggcucauc	2	1	JCK-2
cgugauauugCuucggcucauc	1	1	JCK-2
cgugauauugauucggcucauU	13	1	LT-2
cgugauauugauucggcucauc	137	0	LCK-3
cgugauauugauucggcucauU	1	1	JCK-1
cNugauauugauucggcucauc	1	1	JCK-1
cgugCuauugauucggcucauc	1	1	LT-3
cgugauauugauucggcucaucu	4	0	JCK-3
cgugauauugauucggcucaucu	3	0	JCK-2
cgugauauugauucggcucaucu	15	0	LCK-3
cgugauauugCuucggcucaucu	1	1	LT-1
cgugauauugauucggcucaucA	2	1	LT-1
cgugauauugauucggcucaucA	3	1	LT-2
cgugauauugauucggcucauAu	1	1	LT-2
cgugauauugauucggcucauUu	9	1	LCK-3
cgugauauugauucggcucauUu	2	1	LT-1
cgugauauugauucggcucaucu	34	0	LT-3
cgugauauugauucggcucauUu	1	1	LT-3
cgugauauugauucggcucaucA	3	1	LT-3
cgugauauugauucggcucaucu	1	0	JCK-1
cgugauauugauucggcucaucA	1	1	LCK-3
cgugauauugauucggcucauUu	6	1	LT-2
cAugauauugauucggcucaucu	1	1	LT-2
cgugauauugauucggcucaucu	17	0	LT-2
cgugauauugauucggcucaucu	30	0	LT-1
cgugauauugauucggcucaucuU	1	1	JCK-2
cgugauauugauucggcucaucuU	1	1	LT-2
cgugauauugauucggcucaucuU	2	1	LT-3
cgugauauugauucggcucaucuUu	1	1	LCK-3
.gugauauugauucggcuca	1	0	LT-2
.gugauauugauucggcucauc	1	0	LT-1
.gugauauugauucggcucauc	2	0	LT-3
cgagccgaaucaauauca	1	0	LT-2
cgagccgaaucaauaucU	2	1	LCK-3
cgagccgaaucaauaucac	1	0	LT-1
cgagccgaaucaauaucacu	5	0	LT-2
cgagccgaaucaauaucacu	2	0	LT-3
cgagccgaaucaauaucacu	6	0	LT-1
cgagccgaaucaauaucacuU	1	1	LT-1
